# Development and validation of a smartphone image-based app for dietary intake assessment among Palestinian undergraduates

**DOI:** 10.1038/s41598-022-19545-2

**Published:** 2022-09-14

**Authors:** Sarah Hattab, Manal Badrasawi, Ola Anabtawi, Souzan Zidan

**Affiliations:** Nutrition and Food Technology Department, Faculty of Agriculture and Veterinary Medicine, An-Najah National University, Tulkarm, West Bank, PO. Box: 7, Nablus, Palestine

**Keywords:** Lifestyle modification, Health care, Nutrition

## Abstract

Accurate dietary assessment is required in a variety of research fields and clinical settings. Image-based dietary assessment using smartphones applications offer the opportunity to reduce both researcher and participant burden compared to traditional dietary assessment methods. The current study, conducted in Palestine, aimed to design an image-based dietary assessment application, to assess the relative validity of the application as a dietary assessment tool for energy and macronutrient intake using the 3-Day Food Record (3-DFR) as a reference method, and to test its usability among a sample of Palestinian university students. The development of a smartphone application (Ghithaona) designed to assess energy and macronutrient intake is reported. The application validity was tested among a sample of Palestinian undergraduates from An-Najah National University. Participants recorded their dietary intake using the Ghithaona application over 2 consecutive days and 1 weekend day. Intake from the Ghithaona application were compared to intake collected from 3-DFR, taken on 2 consecutive weekdays and 1 weekend day, in the second week following the Ghithaona application. At the end of the study, participants completed an exit survey to test assess application usability and to identify barriers to its use. Mean differences in energy, and macronutrients intake were evaluated between the methods using paired t-tests or Wilcoxon signed-rank tests. Agreement between methods was ascertained using Pearson correlations and Bland–Altman plots. The Ghithaona application took 6 months to develop. The validation test was completed by 70 participants with a mean age of 21.0 ± 2.1 years. No significant differences were found between the two methods for mean intakes of energy or macronutrients (*p* > 0.05). Significant correlations between the two methods were observed for energy, and all macronutrients (*r* = 0.261–0.58, *p* ≤ 0.05). Bland–Altman plots confirmed wide limits of agreement between the methods with no systematic bias. According to the exit survey, it was found that majority of participants strongly agreed and agreed that the application saves time (94.2%), helps the participant to pay attention to their dietary habits (87.2%), and is easy to use (78.6%). The Ghithaona application showed relative validity for assessment of nutrient intake of Palestinian undergraduates.

## Introduction

Since before the global COVID-19 pandemic, non-communicable diseases (NCDs) had risen significantly across the world, posing a serious public health issue in developing and developed countries alike. The “epidemiologic transition” from infectious diseases to NCDs in developing nations is associated with several risk factors, mostly related to economic and social developments^1,2^. According to Palestinian health annual report-2020, cardiovascular diseases was the first leading cause of death by 24.7%, followed by diabetes mellitus by 14.6%, then cancer by 14.1%^3^. Many of these were conditions related to dietary intake^3^. Although data analysis from the Palestinian Central Bureau of Statistics shows that young adults have a significantly lower prevalence of diabetes, CVD, and cancer than adults^4^, it is established that dietary habits among adolescents and young adults are associated with adulthood chronic disease risk^5^.

Following a healthy dietary habits and lifestyle could therefore be protective, improving health and increasing life expectancy, by preventing NCDs^6^. Specifically, the Mediterranean diet (MD), which includes high intake of extra virgin olive oil, legumes, cereals, fish, vegetables, and fruits; mild consumption of eggs, red wine, milk and dairy products, and a low consumption of red meat and animal fats, exemplifies a part of preventing NCDs^7^.

Gathering dietary intake data is paramount in nutritional epidemiology, as it supports an understanding of diet-disease relationships. However, dietary intake assessment, especially daily dietary intake, still pose a technical problem for both nutritionists and researchers^8^. Determinants of dietary assessment have been well authenticated and differ by the selected method. Scientists usually use traditional methods in assessing dietary consumption: 24-h recall, food frequency questionnaire, and the 3-day food diary (3DFD)^9^. However, these traditional methods are fraught with many methodological issues which make evaluating nutritional intake highly complicated. This has the prospect to result in misinterpreting associations between dietary intake and health status and reduces the ability to evaluate the effectiveness of dietary interventions^8,10^.

Many recent studies in epidemiology and nutrition have therefore focused on the development of accurate assessment methods. Recent research has concentrated on harnessing technology to address existing methodological faults^9^. Technological solutions have the capability to standardize and automate coding, ameliorate communication and commitment, improve data quality, and minimize participant and researcher burden^11,12^. Accordingly, these characteristics can also lower the expenses related to nutritional research. Of particular interest is the use of mobile phones in assisting dietary assessment^9^.

The development of smartphones has resulted in the proliferation of smartphone software applications, which are programs able to run on these mobile apparatus. Form public health perspective, these smartphone applications represent a more convenient way of collecting nutritional intake data than traditional paper-based food records^13^. There is emerging evidence that employing smartphone technology apps for improving the quality of dietary assessment is acceptable, feasible and preferred^14–19^. The portable and social acceptable based food *dietary* apps can be used at the time of eating, decreasing the possible mistakes associated with recalling dietary consumption afterwards^14^. Ji et al.^15^ developed and validated a smartphone app to assess the diet intake among a small sample of Canadians, which was recommended to be used with more larger populations. In Malaysia, it was also reported that using smart apps in dietary assessment among young university students was a well-accepted and valid method^16^. The same findings were reported in other studies conducted among specific populations such as type 2 DM^17^ children with type 1 DM^18^ and pregnant women^19^. Nevertheless, it is important to consider the cultural differences in ingredients, meal presentations, cooking methods and consumption style when using the smart apps in dietary assessment.

Accordingly, in Palestine smartphone applications can be used to facilitate data collection process, especially considering that 86.2% of Palestinian families have smartphones^20^.

Current dietary assessment research in Palestine mainly depends on dietary recall, which is known to be time-consuming, to require training of personnel, and is prone to bias in the estimation of food portion size^21^. This calls for the use of methods which can overcome these difficulties and biases, of which photographic records is one. Although photo-based apps are increasingly used globally^22,23^. They are not tailored to the Palestinian context which has its specific food culture, items and ingredients.

Although college students typically suffer less of the burden of chronic diseases, they are commonly chosen for pilot studies as they tend to be highly engaged, increasing the chance of a high response rate^24^. College students are also generally higher-intensity users of smartphones and new technologies and are better-connected to the internet than the older population, making them an ideal target audience for interventions which require high engagement with technology^25^.

Therefore, we embarked on building a smartphone application to assist university students in assessing their dietary intake easily, as well as, the assessment of dietary intake, especially energy intake, affordable for everyone. This paper aimed to: (1) describe the process of developing a smartphone application, (2) assess the relative validity of the application against the 3DFD, and (3) test the usability of the dietary assessment application by piloting it among a sample of Palestinian university students.

## Methods

### Application development

A multidisciplinary team including nutritionists and software engineers worked together to develop and design a dietary assessment application, Ghithaona. Ghithaona is considered a *native* application (Android system) and uses a firebase real-time database to store the collected data. Our application provides three key functions: (1) creating personal account, (2) logging consumed foods or drinks for any desired duration (e.g., one day, two days, one week, one month, etc.) by clicking on food images, and (3) emailing a professional nutritionist if the user has any inquiry. Throughout the development and evaluation processes, the evaluators provided comments and feedback on the categorization of food items and presentation of portion sizes, which was estimated using household measurements, relying on the Palestinian style and habitual food consumption. For example in food item categorization, mayonnaise was originally classified as an oil and fat, but it was recommended to reclassify it to miscellaneous group. In regard to the presentation of portion size; traditional cereals were presented in three different portion sizes; then it was suggested to add an additional two portions to create a total of five portion sizes for each cereal. Another example, traditional cheese was presented in two portion sizes, then it was suggested to use different shapes and cuts for each portion. In general, most foods’ portion sizes ranged from three to five sizes.

The food database of Ghithaona was sourced from the database of the “Palestinian Food Atlas Project”, which included 368 food items and included nutritional information about energy, carbohydrate, fat, and protein^26^. For every food item retrieved from the database, the images, and amounts for a standard serving size (e.g., 1 cup or 1 small bowl) were archived.

Our application was designed to calculate the Estimated Energy Requirement (EER) which is defined as the medium energy intake that sustains energy balance in normal weight, healthy persons^27^. The EER is calculated using the equation developed by the National Academy of Sciences, Institute of Medicine, and the Food and Nutrition Board, using the user’s height, current weight, age, and physical activity Coefficients^27^.

Once the application was developed, a content validity was done by conducting an evaluation session with twelve experts from four different disciplines (e.g., nutritionists (5), academic staff (3), researchers (3), and IT expert (1)).

### Participants

Participants were university students selected from An-Najah National University in Nablus, Palestine during March 2021 and September 2021. Eligibility criteria included being an undergraduate university student, having a smartphone with an Android operating system, and being able to download the Ghithaona application without assistance. Exclusion criteria included recent gastrointestinal surgery with dietary restriction, presently following a therapeutic diet or weight loss diet, history of an eating disorder, and inability to understand written Arabic. The study protocol was approved by the Institutional Review Board at An-Najah National University (Ref: Mas Oct. 2020/4). Written informed consent was obtained from each participant prior to enrollment.

The sample size was calculated using MEDCALC software for a method comparison study, using the Bland–Altman plot. The following inputs were used: type one error 0.05, type two 0.1, expected standard deviation of differences of 50, expected mean difference of total calories intake of 200^15^. The required sample size result was 70; to account for possible loss of follow-up, the sample was increased to 80 participants.

### Procedure

Each participant met with a researcher (HS) at An-Najah National University. Participants were asked to fill in a short sociodemographic questionnaire with questions related to age, gender, academic year, place of residence, monthly income, physical activity (minutes/day), and the use of dietary supplements; if yes (the type and the dosage). Participant weight and height were self-reported. Body mass index was calculated and classified according to WHO cut off points^24^. In order to decrease the burden on participants and to ease their follow-up, participants were required to record their dietary intake, consumed routinely, using the Ghithaona application through the first week, then they were required to use 3-DFR for the second week. Both Ghithaona and 3-DFR recordings needed to include 2 successive weekdays and 1 weekend day. Participants were given guidance on how to select and enter food items and weights using Ghithaona application virtually via Zoom. Each participant was also instructed and trained on how to estimate portion sizes using household measurement tools, and standard measuring cups. In addition, they were taught how to measure the remaining parts of food, and then to record their dietary intake in 3-DFR. Each participant were given a food diary booklet containing all the required instructions and household measurement photos to guide them in recording their dietary intake using 3-DFR. A total of 80 university students expressed an interest to participate in the study and received an email that contained a link of the Ghithaona download page (http://descargarparaandroid.com/apk/ps.swift.ourfood). Participants received daily WhatsApp messages from researchers to help them keep track of the specific days they should record their dietary intakes using both methods.

### 3-Day Food Record (3-DFR)

All students were given a hard-copy of three pages of the 3-DFR to document their diet (Supplementary File 1). They were given instructions how to complete 3-DFR. They were then asked to estimate and document the details of all main meals and snacks they consumed including the methods used in cooking, the size of the eaten food, and any add-ons (such as dressings, herbs, spices, jam, etc.). If eating meals outside the home (e.g., in restaurants), students were required to document the name of restaurant as well as the name of the meals consumed. All 3-DFR responses were coded to maintain confidentially.

### Nutrient analyses

All foods recorded in 3-DFR were reviewed by a trained researcher. Afterward, the 3-day average nutrient intake for each participant was computed by NutriSurvey software 2007 (EBISpro, Germany) for total energy (kcal), carbohydrate (g), protein (g), and fat (g). As no updated nutritional database has been collected for some Arabic foods, we used the US Department of Agriculture (USDA) nutrient database^25^, NutriSurvey software, and other local and regional food composition databases^26–29^. The data was then exported to SPSS 23 for statistical analysis.

The food records from the Ghithaona application were also exported precisely as they were entered by the participant (Application-participant). A trained researcher then reviewed each food record for missing items and exported these data for analysis to Excel. The nutrition assessment and recommendations were sent to the participants by a research dietitian after the reports were corrected and reviewed by the dietitian.

### Exit survey

Participants were sent a link to an online survey during the course of the study. This survey includes 19 statements with a 5-item rating scale (extremely agree, agree, neutral, disagree, and extremely disagree). The survey aimed to examine the usability of using Ghithaona application among participants. The content of the survey was validated by five experts who were previously involved in the focus discussion group. The results of reliability using SPSS software revealed an acceptable Cronbach alpha value of 0.69.

### Statistical analysis

The normality of distribution of variables: total calories and macronutrients intake from the application and the 3-DFR were assessed graphically and via the Shapiro–Wilk test. Descriptive statistics were performed for sociodemographic variables, as well as the results of the survey. The extreme values were specified statistically, and were identified using a box plot diagram (Whisker's plot), and then the values labelled as stars were removed. Mean or median intakes of energy and macronutrients from 3-DFR and 3-days of the Ghithaona application were calculated and differences determined using paired *t*-tests (normally distributed data for carbohydrate) or Wilcoxon signed-rank test (skewed data for energy, protein, and fat). Bland–Altman plots^28^ were presented to assess the agreement between Ghithaona application and 3-DFR for energy, and macronutrients. Correlations between Ghithaona application and 3-DFR were assessed using Pearson correlation. SPSS Statistics 23 was used to conduct all statistical analyses, and p-values < 0.05 were considered statistically significant.

### Ethics approval and consent to participants

This project acquired an ethical approval from the Institution review Board for Ethical approval from An-Najah National University, Institutional review board ethical committee (Ref: Mas Oct. 2020/4). The research procedures were conducted in accordance with the principle expressed in the Declaration of Helsinki. The consent of participation was taken from the participants before the data collection.

### Informed consent

Informed consent was obtained from all participants of this study.

## Results

### Application development

The application took 6 months to build including creation of the databases, development of the design with a software engineer, and validation of the application. The development process involved professionals from the fields of nutrition and dietetics, and information technology. A total of 368 food items were included in the application as follows: appetizers (46 items), beverages (10), breads (12), dairy products (11 items), desserts (55 items), fats (9 items), fruits (25 items), grains (9 items), legumes (11 items), main meals (83 items), meats (7 items), nuts and seeds (11 items), salads (19 items), salty snacks (9 items), vegetables (26 items), and miscellaneous (9 items), ready to eat items (e.g., chocolate, chips, candies) (16 items). Moreover, the name of each food item were typed and its' portion sizes, which was estimated using household measurements tools, were presented as photos. All comments and suggestions provided by the professionals have been properly considered.

**Figure 1:**
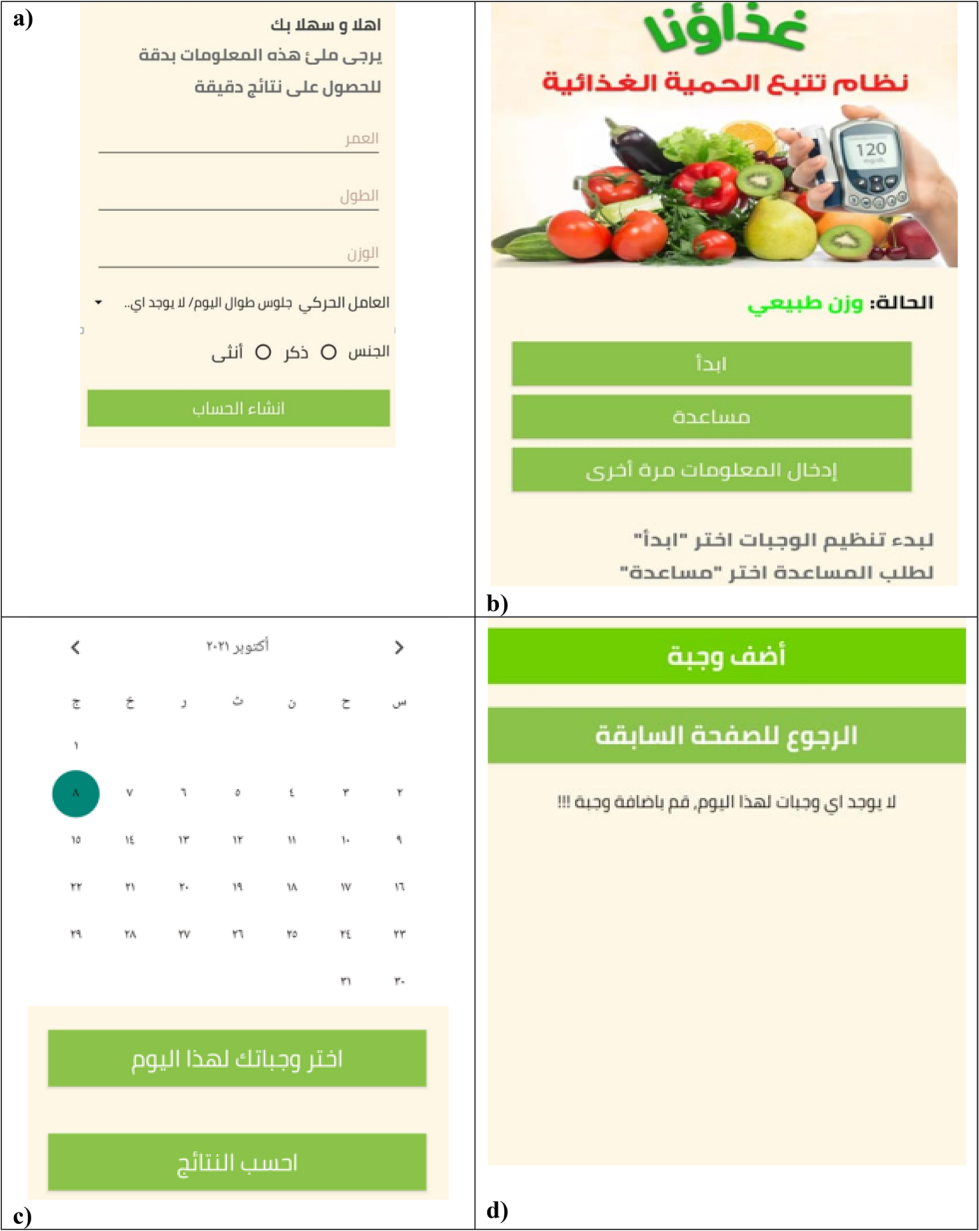

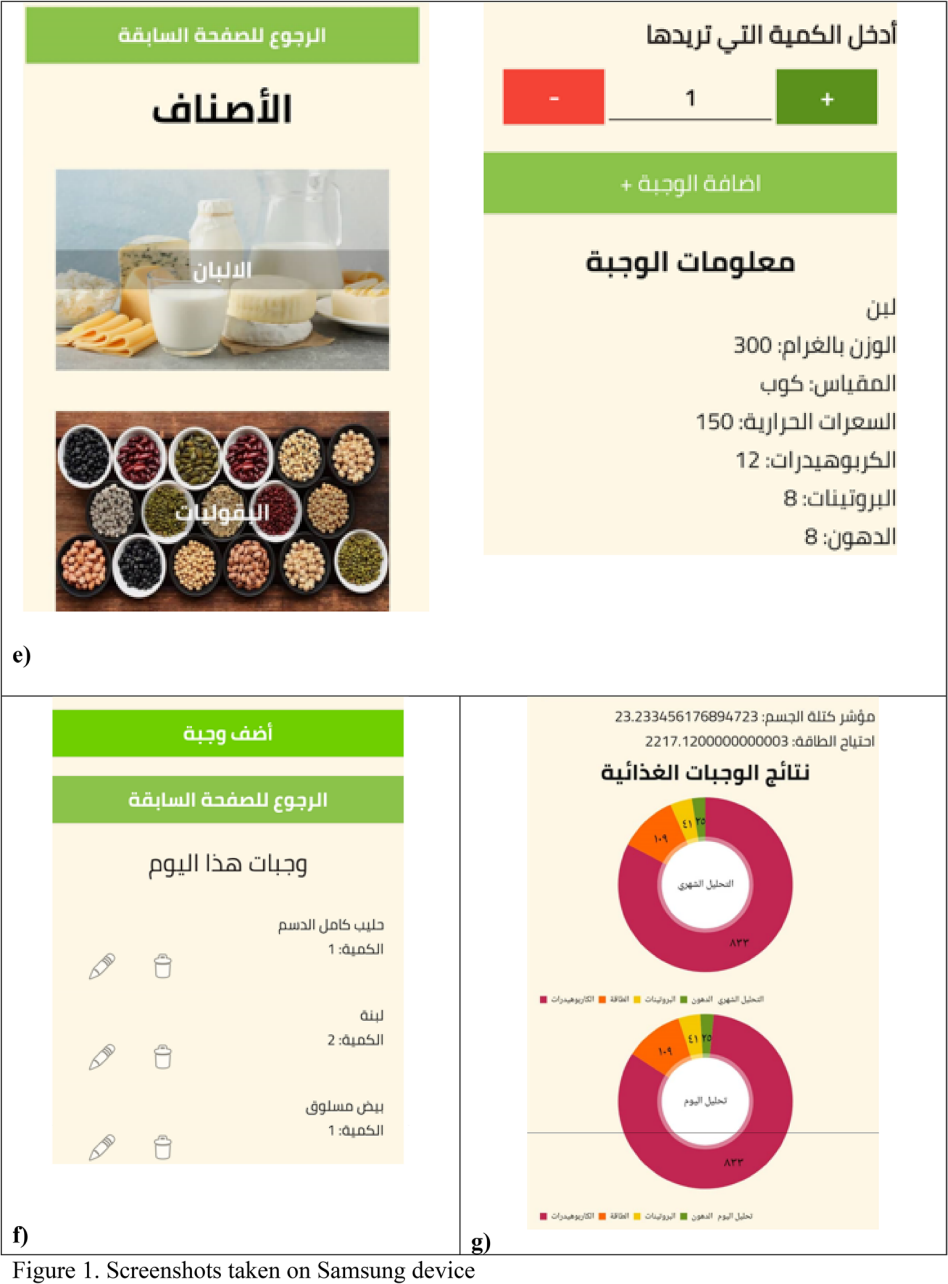
Screenshots taken on Samsung device.

Figure 1a shows the user interface design where each user is required to enter his or her age, sex, weight, height, and physical activity level, which is estimated by the nutritionist based on several questions-related to physical activity. BMI is calculated by dividing weight (kg) by the square of height (m^2^). Figure 1b shows the main page after the user logging in which consists of three interface categories: start, help, and re-entering the information. Note, the user can request to add a new food, not available in the application database, by clicking on button “help”, thereafter a notification will be sent to the researchers, who are responsible for modifying the food database.

After pressing the start button, another screen, shown in Fig. 1c, appears, consisting of a calendar to choose the date of the day they started to record their dietary intake, and two buttons; (1) select your meals for today, and (2) calculate the dietary intake.

By clicking on button “Select your meals for today”, a screen containing “Add a meal” button and “Return to the previous page” button, will be displayed as illustrated in Fig. 1d. Then, the user selects “Add a meal” to select meals.

Screens displayed in Fig. 1e allow user to choose photos that represent a range of different portion sizes of their consumed foods or drinks, then, user can determine the amounts of their consumed foods or drinks, as there is different options of frequencies (e.g., ½, 1, 1 ½, etc.) After user log all the foods and supplements, they can review the logged foods and beverages. User can also delete or edit some foods/beverages before submission if they desire (Fig. 1f).

Once a user completes logging their food intake, they click on the "Calculate the results" button, is found in the screen displayed in Fig. 1c. Dietary intake data from foods and beverage are automatically forwarded to the server and analyzed to provide dietary feedback. User are provided tailored diet feedback on daily energy and macronutrient intake, as the green color represents total fat intake per day, yellow color represents total protein intaked per day, orange color represents total energy intake per day, and pink color represents total carbohydrate intake per day (Fig. 1g).

### Participant recruitment

Participants were recruited from An-Najah National University. A total of 80 participants were invited to join the study and provide their consent. 70 participants were included in the final analysis. The remaining participants were excluded from analysis because they did not complete the 3-DFR (4), wrongly estimated the portion size of meals (i.e. they assessed their portion sizes using household measurements rather than the training-recommended ones) (4), and were outliers of extreme values (2).

### Participants’ recruitment and characteristics

Participants were recruited from An-Najah National University. A total of 80 participants were invited to join the study and provide their consent. 70 participants were included in the final analysis. The remaining participants were excluded from analysis because they did not complete the 3-DFR (4), wrongly estimated the portion size of meals (i.e. they assessed their portion sizes using household measurements rather than the training-recommended ones) (4), and were outliers of extreme values (2).

And concerning participants’ characteristics, the student sample was composed of 60 (85.7%) females and 10 (14.3%) males. The mean age of participants was 21.0 ± 2.1 years, ranged from 18 to 25 years old. Only one participant (1.4%) was in the sixth academic year. Most participants (60.0%) were residents either in camps or villages and nearly half of them (45.7%) had a family income ranges between 1500 and 3000 NIS per month. The majority of participants reported not being interested in exercising, while only 12 participants (17.1%) exercised regularly. Our analysis also revealed that nearly half of participants (52.9%) had normal weight, whereas five participants (7.1%) were obese. Moreover, there was no statistical difference between genders in terms of their general characteristics (*p* > 0.05) as shown in Table 1.

**Table 1:** Participant characteristics according to their gender presented in numbers (n) and percentages (%).

Variables		Males (n = 10)		Females (n = 60)		Total (n = 70)		*p*-value
		n	%	n	%	n	%	
Academic year	1st	3	30.0	7	11.7	10	14.3	0.057
	2nd	4	40.0	21	35.0	25	35.7	
	3rd	0	0.0	11	18.3	11	15.7	
	4th	2	20.0	18	30.0	20	28.6	
	5th	0	0.0	3	5.0	3	4.3	
	6th	1	10.0	0	0.0	1	1.4	
Place of residence	City	4	40.0	24	40.0	28	40.0	0.641
	Camp/village	6	60.0	36	60.0	42	60.0	
Monthly income	< 1500 NIS	0	0.0	1	1.7	1	1.4	0.054
	1500–3000 NIS	1	10.0	31	51.7	32	45.7	
	> 3000 NIS	9	90.0	28	46.7	37	52.9	
Exercising	Not interested	7	70.0	38	63.3	45	64.3	0.750
	Regularly^a^	2	20.0	10	16.7	12	17.1	
	Irregularly	1	10.0	12	20.0	13	18.6	
BMI categories	Underweight	2	20.0	9	15.0	11	15.7	0.472
	Normal weight	4	40.0	33	55.0	37	52.9	
	Overweight	4	40.0	13	21.7	17	24.3	
	Obesity	0	0.0	5	8.3	5	7.1	

Sociodemographic characteristics, lifestyle habits, and body weight status.

NIS, new Israeli Shekel.

^a^30 min of moderate-intensity exercise at least 5 days per week.

#### Criterion validity of the Ghithaona application

There were statistically significant correlations between Ghithaona application and the 3-DFR method for energy, carbohydrates, protein, and fat (correlations ranging from 0.261 to 0.582; p ≤ 0.05) as shown in Table 2. The differences between the methods were not significantly different (p > 0.05) for energy, carbohydrates, protein, and fat. The Ghithaona application yielded lower intakes for nutrients protein and fat than the 3-DFR method; with intakes approximately 16% lower for protein, and 4% lower for fat. On the otherhand, the Ghithaona application yielded higher intakes for nutrients energy and carbohydrate than the 3-DFR method, with intakes approximately 7% higher for energy, and 6% higher for carbohydrate.

**Table 2:** Differences in nutrient intakes recorded by the 3-DFR and Ghithaona application.

Nutrients	Ghithaona application	3-DFR	Correlation coefficient (r)	Difference	Limits of Agreement (LOA)^&^		p-value**
	Mean (SD)	Mean (SD)		Mean (SD)	Lower	Upper	
Energy (kcal/day)	1684 (622)	1560 (520)	0.582*	− 124 (530)	− 1163	914	0.100
Carbohydrate (g/day)	190 (71)	179 (65)	0.450*	− 10 (72)	− 151	130	0.229
Protein (g/day)	69 (33)	82 (83)	0.261*	13 (81)	− 146	172	0.426
Fat (g/day)	72 (35)	75 (38)	0.551*	3 (35)	− 65	71	0.495

SD = Standard Deviation.

*p ≤ 0.05 using Pearson correlation.

**p-value is the significance level between two methods using Wilcoxon signed-rank test (skewed data for energy, protein, and fat) or paired *t*-tests (normally distributed data for carbohydrate).

^&^Lower and upper Limits of Agreement (LOA) (mean difference ± 1.96 SD).

For energy intake, the mean difference between the Ghithaona application and the 3-DFR method was not significant; the mean energy intake from the 3-DFR was 1560 ± 520 kcal/day while from the application was 1684 ± 622 kcal/day (p > 0.05), with (− 124 kcal/day; 95% Confidence Interval (CI) for bias =  − 250 to − 2 kcal/day) (p > 0.05). For random error, the 95% lower and upper Limits of Agreement (LOA) between the two methods for energy intake ranged from − 1163 to 914 kcal/day.

Similarly, the mean difference for carbohydrate, protein and fat intakes were not statistically significant. The mean carbohydrate intake from the 3-DFR was 179 ± 65 g/day, from the application 190 ± 71 g/day (− 10 g/day). The mean protein intake from the 3-DFR was 82 ± 83 g/day, from the application 69 ± 33 g/day (13 g/d). The mean fat intake from the 3-DFR was 75 ± 38 g/day, from the application 72 ± 35 g/day (3 g/day).

The 95% LOA for each of these were narrow (− 151 to 130 g/day for carbohydrates, -146 to 172 g/day for protein, and − 51 to 71 g/day for fat as illustrated in Table 2.

**Figure 2:**
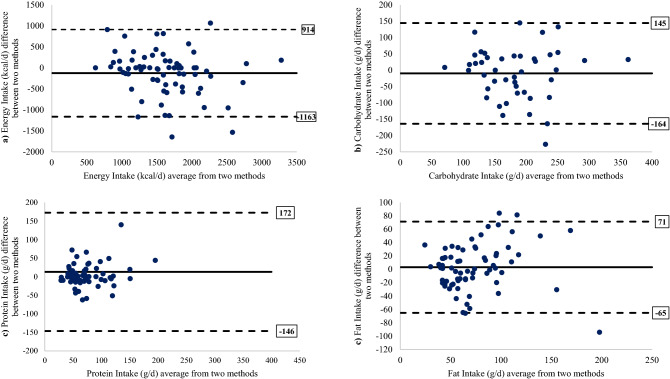
Bland–Altman plots showing mean difference (3-DFR—Ghithaona app, solid line) vs. mean intakes ((3-DFR + Ghithaona app)/2) for (**a**) energy; (**b**) carbohydrate; (**c**) protein; (**d**) fat. The dotted lines indicate the 95% limits of agreement (SD 1.96).

The Bland–Altman plot (Fig. 2) is a scatter plot in which the Y axis shows the difference between the two measurements (from 3-DFR and the Application) and the X axis represents the average of these measures. As recommended, more than 95% of the data points lie within ± 2 standard deviations. The Bland–Altman plots (Fig. 2) for energy, carbohydrate, protein, and fat also illustrate that data for most participants were within the LOA with few outliers, and there was no apparent proportional bias.

### Perceived usability by using Ghithaona application

The analysis of usability survey showed that nearly half of participants strongly agreed that the application is easy to use (48.6%), saves time (47.1%), and helps the participant to pay attention to their dietary habits (52.9%). Furthermore, nearly half of participants agreed that they would like to use this application on continuous basis (50.0%), they felt confident while using the application and believed that most people will learn to use this application very quickly (47.1%). Moreover, half of them agreed that the application can be an alternative for traditional dietary assessment methods (44.3%), decrease participant burden (48.6%), determine the amount of consumed food accurately (52.9%), and help the participant to remember the consumed food items (57.1%). Thirty-one participants (44.3%) also strongly disagreed with the following statement “the application is too cumbersome to use”, however, thirty-three participants (47.1%) disagreed with the following statement “I found that the different functions of this application are well integrated”. Full details regarding perceived usability of the Ghithaona application can be found in Table 3.

**Table 3:** Participants’ perceived usability of using the Ghithaona application for dietary assessment (n = 70).

Perceived usability	Strongly disagree	Disagree	Neutral	Agree	Strongly agree
	n (%)	n (%)	n (%)	n (%)	n (%)
It was found that using the application is easier compared to using pen-paper methods	5 (7.1)	0 (0.0)	10 (14.3)	21 (30)	34 (48.6)
I found that using the application saves time	0 (0.0)	0 (0.0)	4 (5.7)	33 (47)	33 (47.1)
I found that using the application decreases participant burden	0 (0.0)	1 (1.4)	6 (8.6)	34 (48.6)	29 (41.4)
I found that the application is accurate in determining the amounts of consumed foods	0 (0.0)	2 (2.9)	4 (5.7)	37 (52.9)	27 (38.6)
I found that using the application helped me to remember the consumed food items	0 (0.0)	1 (1.4)	11 (15.7)	40 (57.1)	18 (25.7)
I found that the application contributes in rapid analysis of data	0 (0.0)	1 (1.4)	1 (1.4)	24 (34.3)	44 (62.9)
I found that using the application helped me to pay attention to dietary habits	1 (1.4)	0 (0.0)	8 (11.4)	24 (34.3)	37 (52.9)
I found that the application presented many healthy food options from various food groups	0 (0.0)	4 (5.7)	14 (20.0)	27 (38.6)	25 (35.7)
I found that the application is an alternative for pen-paper methods, as the application provides special features	0 (0.0)	2 (2.9)	10 (14.3)	31 (44.3)	27 (38.6)
I found that the application presented a comprehensive list of food items	0 (0.0)	18 (25.7)	17 (24.3)	28 (40.0)	7 (10.0)
I think I would like to use this application on a continuous basis	0 (0.0)	5 (7.1)	10 (14.3)	35 (50.0)	20 (28.6)
I found that the application is unnecessarily complex	19 (27.1)	34 (48.6)	12 (17.1)	4 (5.7)	1 (1.4)
I thought it is easy to use this application	3 (4.3)	4 (5.7)	0 (0.0)	47 (67.1)	16 (22.9)
I think I need technical support to be able to use this application	22 (31.4)	24 (34.3)	17 (24.3)	2 (2.9)	5 (7.1)
I found that the different functions of this application are well integrated	0 (0.0)	3 (4.3)	11 (15.7)	45 (64.3)	11 (15.7)
I think there is a lot of inconsistency in this application	24 (34.3)	33 (47.1)	4 (5.7)	2 (2.9)	7 (10.0)
I think most people will quickly learn to use this application	4 (5.7)	0 (0.0)	3 (4.3)	33 (47.1)	30 (42.9)
I found the application is too cumbersome to use	31 (44.3)	18 (25.7)	6 (8.6)	6 (8.6)	9 (12.9)
I felt very confident while using the application	0 (0.0)	2 (2.9)	13 (18.6)	33 (47.1)	22 (31.4)
I needed to learn a lot of things before I could use this application	20 (28.6)	27 (38.6)	8 (11.4)	5 (7.1)	10 (14.3)

## Discussion

The main purpose of the current study is to develop a smartphone app that provides a foods and drinks database, assessment of food consumption, and personalized feedback. The study also aims to establish the validity of the app relative to the 3-DFR, using a sample of Palestinian undergraduates at An-Najah National University. The Ghithaona app was found to provide acceptable data and to analyze data precisely. It was also demonstrated that the app results are comparable to the conventional 3-DFR method to assess dietary intake among sample of Palestinian undergraduates, since mean intakes of total energy and macronutrients (carbohydrate, protein, and fat) were analogous in both methods. The correlation coefficients between the two methods ranged from 0.261 to 0.582. Furthermore, Bland–Altman plots showed an acceptable level of agreement between the Ghithaona app and the 3-DFR for total energy and all macronutrients without bias, and most data dots fall within two standard deviations of the mean (narrow LAO).

These findings are in accordance with several validation studies^14,29–31^. Rangan et al.^29^ found no significant differences between 24-h recall and mobile phone application (e-DIA) for mean dietary intakes, as well as, Bland–Altman plots demonstrated robust agreement between the methods with minimal bias. In a previous study by Carter et al.^30^, it was demonstrated that the average difference between the two methods (My Meal Mate vs. 24-h dietary recalls) was not significant for both energy and macronutrients. Comparison between 24-h dietary recalls and food records gathered using a mobile phone application (My Meal Mate) also identified consistent findings with moderate to strong correlations between the two methods (7-day PDA vs 24-h dietary recalls); there were no significant differences between average intakes of total energy and all macronutrients, and no obvious bias using Bland–Altman analysis^31^. Ambrosini and his colleagues found that the mean difference for all intakes except alcohol between the 24-h dietary recalls and the mobile application “Easy Diet Diary application” was not significant^14^. This proposes that the findings of the current study are comparable with former studies examining the use of image-based dietary records in undergraduates, and have provided support for their utilization.

Checking the validity of smartphone applications designed to assess dietary intake may be challenging in terms of selecting an appropriate reference method, because the traditional method itself has constraints that challenge its use as a reference measure for assessing nutrient intake. However, if smartphone applications have outstanding performance in comparison to traditional pen-to-paper methods, this requires examination by testing their prediction ability of health consequences in large-scale studies^32^.

The Ghithaona application improves data quality by offering a wide range of food options, as well as, it provides a variety of options for different portion sizes for the same food. Moreover, as the current application has the capability to calculate the dietary intake automatically, this means that it can minimize the time in comparison to traditional methods which is known to be time-consuming. From future prospective, this application will be used as baseline of dietary intake data, which will then be correlated with a many health issues (e.g., diabetes mellitus, cardiovascular disease, etc.). It is important to highlight that the dietary record approach in this application used a photo-based selection, as participants selected their intake according to photos of food items, with multiple photos for different portion sizes. This wasn’t the case in previous studies, where intake was only selected by written food items and their gram weights. Additionally, the Ghithaona application included photos of food items that are presented according to meal presentations, cooking methods, and ways of consumption in the Palestinian culture. This was a limitation for Palestinian users using other international applications. These factors have ensured a structured and feasible dietary record for participants. Besides, the Ghithaona application was well-received by university students in this study; nearly half of participants strongly agreed that the application is easy to use (48.6%), and saves time (47.1%). That the Ghithaona application was well-received may be a consideration for researchers working in the field of adolescents’ nutrition, as a possible way for dietary assessment which has showed acceptability among this group of undergraduates.

There are some limitations to this study. Firstly, the study only included participants from one university. These results are therefore not representative for all undergraduates in Palestinian universities. Secondly, prior research has asserted that three days may be enough for assessing mean energy intakes of individuals but may not be long enough to precisely estimate intake of macronutrients^33^. Additional days of recording may accordingly have been desirable. Thirdly, we did not assess micronutrient intake using the application. Fourthly, there is no published Palestinian food composition tables, therefore we used the Palestinian Food Atlas which in turn used food composition tables for Bahrain^26^, and Lebanon^27^, and exchange lists for Jordan traditional dishes^28^ and desserts^29^, as well as NutriSurvey software and US Department of Agriculture (USDA) nutrient database^25^, in order to estimate the energy and macronutrient content of foods. Finally, the majority of our sample were female, as females were expected to be more concerned about nutrition-related issues compared to males. Therefore, we suggest that future studies should use purposive sampling method to select its sample in order to have a balanced gender composition.

On the other hand, the current study has several strengths. Firstly, this study in its development of an image-based dietary assessment application and its assessment of its validity, is the first of its kind in Palestine. Secondly, undergraduates were able to download the Ghithaona application on their own smart phones from an Android platform, compared to former studies where participants were asked to use an externally-provided “study” apparatus^33^. Ghithaona app is an innovative way to assess the dietary intake of individuals in real time and subsequently does not depend on memory. Another advantage of using this app is that undergraduates are familiar of using smartphones. Moreover, this app can be further developed to become versatile for instance, addressing the dietary needs of individuals with various medical conditions (e.g., hypertension, diabetes, etc.).

## Conclusion

The current study examined the relative validity of Ghithaona, a novel smartphone application, against the 3-DFR among Palestinian undergraduates. Our findings demonstrate that the Ghithaona application saves time according to what reported from participants. Moreover, our application was well-accepted by participants in comparison to conventional pen-to-paper methods. The Ghithaona application may therefore be a useable method to assess dietary intakes among undergraduates. While promising as an alternative dietary assessment method for assessing the dietary intake of Palestinian undergraduates, the smartphone application still requires validation in other age groups as well as in a larger and broader sample size. Future research is required to understand how the application can be upgraded from the user perspective.

## Supplementary Information

Supplementary Information.

## Data Availability

The dataset that support the results and findings of this research are available from the corresponding author, upon request.
