# Supplementary material for: Development and validation of a smartphone image-based app for dietary intake assessment among Palestinian undergraduates
Source: Sci Rep. 2022 Sep 14;12:15467. doi: 10.1038/s41598-022-19545-2 (PMC9472744; doi:10.1038/s41598-022-19545-2)
Supplement: Supplementary file 1 — Supplementary Information. [file 41598_2022_19545_MOESM1_ESM.pdf]

Faculty of Graduate Studies

An- Najah National University

Nablus – Palestine

Development and validation of a smart-phone app to estimate diet intake among university students

Supplementary file 1

Food record booklet

كتيب لتسجيل الطعام

الاسم :	التخصص الجامعي :
العمر :	رقم الهاتف:
الجنس :	بريد الكتروني نشط:
الوزن :	الطول :

تم تصميم هذا الكتيب لأهداف علمية ضمن بحث رسالة ماجستير, تهدف الى تصميم تطبيق على الهواتف الذكية لتطوير عملية تسجيل الطعام و تحليلها. ولذلك صمم هذا الكتيب ليقوم المشارك بالدراسة بتدوين وجباته اليومية الاعتيادية لمدة ثلاث أيام ،نحيطكم علما بان هذه المعلومات ستبقى سرية وفقط ستستخدم لأغراض البحث العلمي.

يحتوي الكتيب على أربعة جداول, الأول : مثال لكيفية التسجيل, الثلاثة الباقية لتسجيل ما يتم تناوله من وجبات رئيسية, وجبات خفيفة, مشروبات, حلويات, وأي شيء آخر. ل ثلاثة ايام مختلفة , يومين عمل و يوم عطلة.

طريقة تسجيل الطعام :

1. ضرورة الانتباه و تسجيل اوقات تناول الطعام .
2. تحديد اسم الوجبة, مثل: وجبة فطور, وجبة غداء, وجبة عشاء, وجبة خفيفة.
3. تسجيل الطعام المستهلك: تفاحة, كوب حليب, رغيف خبز, أرز, قطعة شوكولاته (مع ذكر اسم الشركة).
4. يرجى ذكر اسم الشركات لمنتجات السوق, مع تحديد كميتها بناءا على ما هو مكتوب على عبوة المنتج, و اذا تم استهلاكها كامله, نصفها و هكذا.
5. تقدير الكميات المستهلكة بناءا على جدول معطى فيه صور لادوات منزلية تساعد في تقدير الكميات, في حال وجود اكواب المعايير يرجى استخدامها, ايضا في حال وجود ميزان الطعام يرجى استخدامه للخبز مثلا .
6. ذكر أية اضافات على اصناف الطعام , مثل : ملعقة طحينية, ملعقة عسل , ملعقة زيت زيتون , ملعقة زبدة.

الجدول الأول : مثال توضيحي لكيفية تسجيل الطعام .

الوقت	اسم الوجبة	الوجبة	مكونات الوجبة	كمية الوجبة المتناولة	الإضافات
08:30 صباحا	وجبة خفيفة	كوب حليب<br>نصف كوب شوفان		كوب كبير<br>نصف كوب كبير	معلقه صغيرة عسل.
12:00	الفطور	بيضة مقلية ب زيت, حمص ، خبز اسمر ،بندورة شاي.		2 معلقه كبيرة زيت<br>2 معلقه كبيرة حمص<br>رغيف خبز اسمر (60غم)<br>حبة بندورة<br>كاسة شاي	تم اضافه معلقه ونصف صغيره سكر ابيض لكاسه الشاي
04:00	وجبة خفيفة	تفاح		حبة تفاح وسط	
07:30	الغذاء	مقلوبة مع دجاج<br>لبن رايب<br>سلطة خضار مع بصل.	باذنجان،دجاج ،بطاطا.	2 كفكيره مقلوبة<br>سفينة الدجاج<br>قطعتين بطاطا<br>صحن مجور صغير لبن رايب<br>صحن مجور صغير سلطة	معلقه كبيرة زيت زيتون على صحني السلطة.
09:30	وجبة خفيفة	قهوه عربية<br>قطعة شوكولاته		فنجان صغير<br>نص حبة.	قطعة واحده فقط من شوكولاتة الباونتي.
12:00	وجبة خفيفة	مكسرات مشكلة محمصة<br>عصير فواكه	مانجا	قبضتين يد (كمشتين)<br>نص صحن مجور صغير.<br>كوب كبير	طبيعي بدون اضافات.

كاسه شاي	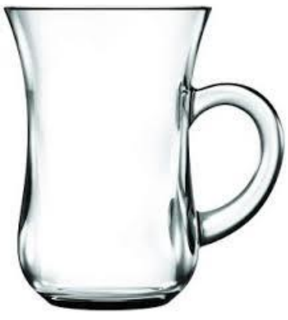
كوب 250 مل (كاسه كبيرة)	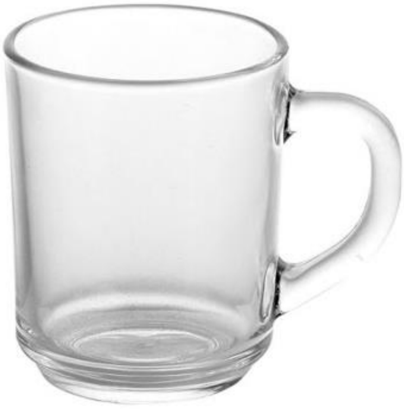
فنجان قهوة	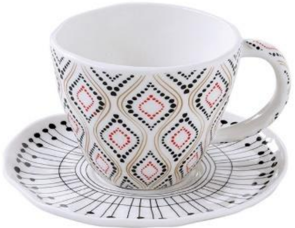

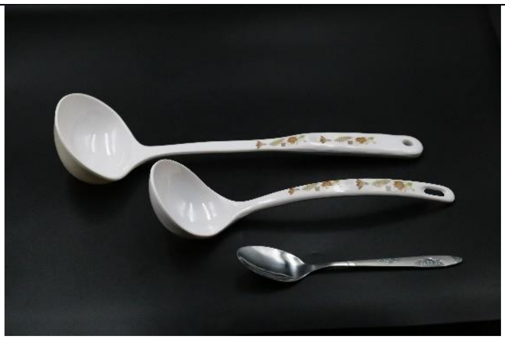	مغرفة مرقه (صغير, كبير)
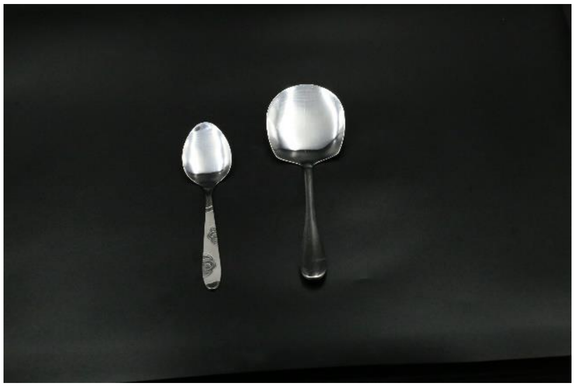	مغرفة رز
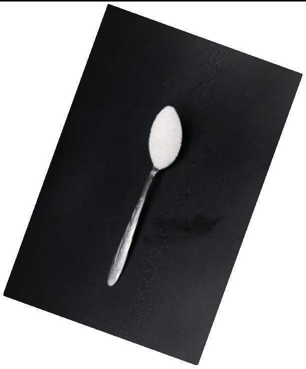	معلقة طعام صغيرة

معلقة طعام كبير	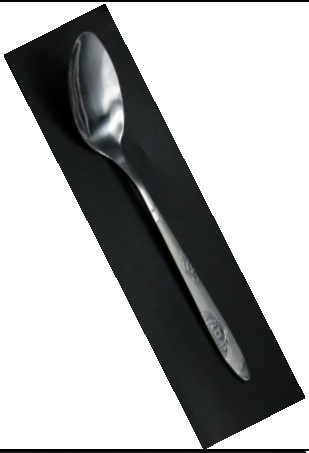
صحن مجور صغير	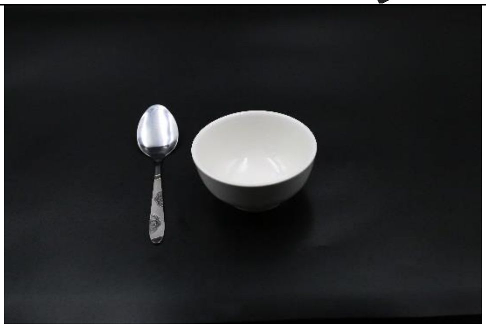
المعايير	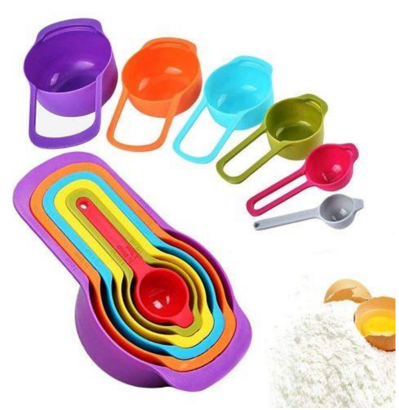

قوائم تسجيل الطعام

اليوم الأول<br>يوم عمل.					
الوقت	اسم الوجبه	الوجبة	مكونات الوجبة	كمية الوجبة المتناولة	الإضافات

اليوم الثاني:

يوم عمل.

الوقت	اسم الوجبه	الوجبة	مكونات الوجبة	كمية الوجبة المتناولة	الإضافات

اليوم الثالث:

يوم اجازة.

الوقت	اسم الوجبه	الوجبة	مكونات الوجبة	كمية الوجبة المتناولة	الإضافات
